# Time-Restricted Eating Combined With Progressive Personalized Lifestyle Modifications for Adolescent Obesity: A 12-Month Case Series

**DOI:** 10.7759/cureus.101493

**Published:** 2026-01-13

**Authors:** Manoj Kumar, Alka Shukla, Rajshri Aishwarya, Asha Pathak, Indu Saxena

**Affiliations:** 1 Physiology, Maharshi Vashishtha Autonomous State Medical College, Rampur, IND; 2 Pediatrics, Maharshi Vashishtha Autonomous State Medical College, Rampur, IND; 3 Medical Education, Uttar Pradesh University of Medical Sciences, Saifai, IND; 4 Pharmacology, Uttar Pradesh University of Medical Sciences, Saifai, IND; 5 Biochemistry, All India Institute of Medical Sciences, Gorakhpur, Gorakhpur, IND

**Keywords:** insulin resistance, lifestyle management, lipid profiles, time-restricted eating, weight loss and obesity

## Abstract

Four adolescents, first cousins (three male, one female) residing in a joint family household, of age 11-18 years, reported with lifestyle-related obesity (established from the Z-scores for age and gender). Eleven out of 12 members of the joint family were suffering from overweight or obesity. One male adolescent (17 years, seven months) had pre-diabetes as evident from the values of fasting plasma glucose and HbA1c. Due to the young age of the patients, the parents were not in favor of initiating pharmacotherapy. A detailed study of the lifestyle of the four patients revealed unhealthy eating habits, a long duration of the eating window, short sleep duration, and a lack of physical activity. Laboratory investigations revealed elevated levels of fasting glucose, total cholesterol, triglycerides, triglyceride-glucose index (TyG), and liver enzymes, indicating insulin resistance.

This case-series (without any control subjects) of one year's duration involved subjects habituated to an unhealthy lifestyle. Lifestyle modifications (LSMs), including time-restricted eating (TRE), were implemented in a slow and progressive manner over a period of two months. Physical measurements and laboratory parameters were noted at zero, six, and twelve months. Care was taken to avoid nutritional deficiencies and to ensure optimum growth.

Anthropometric parameters, fasting glucose, lipid profile, and liver enzymes showed marked improvement after 12 months. All four patients reduced their BMI from the obese to the overweight category. The BMI reduced from 26.4+/- 2.8 kg/m^2^ to 23.5 +/- 0.8 kg/m^2^. The extent of weight loss was different in all four individuals, as they had not observed equal adherence to the recommended LSMs.

Development of healthy lifestyle is crucial for children to avoid weight-related issues when they are older. Unrestricted food intake and long eating windows can lead to weight gain and obesity at an early age. This study shows the importance of early interventions with LSMs in adolescent patients with obesity.

## Introduction

The incidence of obesity in children is increasing in India and throughout the world. According to the Non-Communicable Disease (NCD) Risk Factor Collaboration Report published in 2024 [1], globally, more than 390 million children/adolescents between the ages of five and 19 years were overweight in 2022. The prevalence of overweight and obesity in this age group increased from 8% in 1990 to 20% in 2022. The number of children/adolescents with obesity was 31 million in 1990; it increased more than four times to 160 million in 2022. India has a large population of adolescents: 253 million (20.9%) or one-fifth of the total population [2]. According to the Indian Academy of Pediatrics (IAP), body mass index (BMI) values between the 85th and 95th percentile indicate overweight, while a BMI above the 95th percentile indicates obesity [3]. Almost one-fifth of the population in India comprises adolescents, of whom about 6% are overweight, and about 2% have obesity [4]. The increasing prevalence of overweight and obesity in children is alarming, as children with these conditions usually grow up to become adults with overweight/obesity, and are at a higher risk of developing weight-related morbidities.

Overweight and obesity may result from both genetic and environmental causes [5]. The most common environmental causes are excess calorie intake, insufficient physical exercise, stress and psychological factors, pre-natal and post-natal factors, certain medicines, and environmental pollutants like endocrine-disrupting chemicals. These factors do not work in isolation but have a combined impact on the individual’s weight and health status. Various physical, metabolic, reproductive, and psychosocial conditions result from overweight and obesity, and may increase healthcare expenses [6]. It is difficult to regulate the food intake in case of children/adolescents with obesity, as the nutritional deficiency incurred may affect growth. Obesity medications and bariatric surgeries are considered dangerous by the parents and are often declined. However, carefully monitored lifestyle modifications can help improve the overall health of these patients.

The subjects in this study were the adolescent first cousins (including one pair of siblings) of the 17-year-old male proband, who had recently been diagnosed with prediabetes. The subjects were members of the same joint family, sharing the same kitchen and meals. The parents were against weight-loss surgery and pharmacotherapy. These cousins had already tried different diets for weight loss but had not been successful. This prospective observational case series of one-year duration investigates if time-restricted eating (TRE) and lifestyle modifications result in weight loss in adolescents with obesity.

## Case presentation

The 17-year-old male proband (research participant A) was identified as a prediabetic when his medical fitness was evaluated at the time of joining a graduate para-medical course. His random blood glucose at that time was 188.1 mg/dL (reference value: <140 mg/dL). He subsequently visited the outpatient department of the college for further tests and treatment. He reportedly smoked one to four cigarettes per day (a recently developed habit for peer acceptance), but reported no addiction, and assured he could easily stop smoking. His anthropometric parameters were recorded, and routine laboratory tests were prescribed (results are summarized in Tables 1, 2).

**Table 1:** Anthropometric parameters and blood pressure of the participants before initiating lifestyle modifications

Subject ID	A	B	C	D
Date of study initiation	30. 08. 23	04.09.23	04.09.23	04.09.23
Age (years, months)	17, 7	15, 2	11, 09	11, 05
Gender	M	M	F	M
Height (cm)	172	168	155.5	158
Percentile	50	>50	>90	>90
Weight (kg)	89	75.5	57	62
BMI	30.1	26.8	23.7	24.8
Z-Score	>2 SD	>2 SD	>2 SD	>2 SD
Waist (cm)	105	96.5	87.5	91
Hips (cm)	107	100.5	93	95.5
Waist-hip ratio	0.98	0.96	0.94	0.95
Systolic blood pressure (mmHg) (reference value: <120)	117	110	107	108
Diastolic blood pressure (mmHg) (reference value: <80)	75	72	68	70

**Table 2:** Laboratory parameters of the participants before initiating lifestyle modifications HDL: High-density lipoprotein; LDL: Low-density lipoprotein; A/G ratio: Albumin-to-globulin ratio; AST: Aspartate transaminase; ALT: Alanine transaminase; GGT: Gamma glutamyl transferase; ALP: Alkaline phosphatase; TyG: Triglyceride-glucose index; TBIL: Total bilirubin; DBIL: Direct or conjugated bilirubin.

Parameter	Normal	A	B	C	D
Fasting blood glucose (mg/dL)	60-100 [7]	121.8	103.3	97.7	101.4
2-hour post-prandial blood glucose (mg/dL)	<140	138	131.2	132.1	128.7
Hemoglobin (g/dL)	13-16 (lower in females) [8]	17.2	16	15.4	15.2
Thyroid stimulating hormone (TSH) (mU/L)	1.83-3.6 [9]	3.01	2.09	3.2	2.54
Total cholesterol (mg/dL)	<170 [10]	182.4	178.2	172.6	176.5
HDL-cholesterol (mg/dL)	>45 [10]	37.4	38.6	48.5	47.3
LDL-cholesterol (mg/dL)	<110 [10]	108.2	107.8	93.6	97.5
Total triglycerides (mg/dL)	<90 [10]	184.2	159.2	152.6	158.7
Total protein (g/dL)	6-8 [7]	8.5	8.3	7.8	8.8
Total albumin (g/dL)	3.5-5.0 [7]	4.8	5	4.8	5.1
A/G ratio		1.3	1.5	1.6	1.4
AST (IU/L)	<31 (F) or 35 (M) [7]	52	35	28	30
ALT (IU/L)	<25 (F) or 30 (M) [7]	54	37	25	25
GGT (IU/L)	<26 (F) or 43 (M) [7]	41	38	23	25
ALP (IU/L)	<320 (F) or 390 (M) [7]	328	319	302	316
TyG	<4.49 [11]	5.01	4.85	4.8	4.84
TBIL (mg/dL)	0.1-1.2 [7]	0.83	1.01	0.82	0.78
DBIL (mg/dL)	<0.2 [7]	0.08	0.06	0.06	0.07

The fasting blood glucose (FBG) was 121.8 mg/dL, and his glycated hemoglobin (HbA1c) was 6.2% (normal FBG: 60-100 mg/dL; normal HbA1c: 4.5-5.6% [7]). He was prescribed lifestyle changes, as his parents wished to try metformin only if the lifestyle changes were ineffective. On his subsequent visit, he was accompanied by his three adolescent first cousins. The anthropometric parameters of the patients were recorded at the time of presentation, and are summarized in Table 1. Hemoglobin, FBG, liver function tests (LFT), and lipid profiles were obtained to examine nutritional status and metabolic status. The results are presented in Table 2.

The TSH levels were well within the normal range excluding hypothyroidism. Although patients C and D had heights above the 90th percentile for their age, the parents and other family members of these patients had average heights. All four patients had BMI higher than +2SD for their age and had clinical obesity. Hemoglobin, total protein, and albumin were normal for age and gender. Total serum cholesterol, triglycerides, and low-density lipoprotein (LDL)-cholesterol levels were on the higher side of normal. In the case of patient A, the serum levels of aspartate aminotransferase (AST), alanine aminotransferase (ALT), gamma glutamyl transferase (GGT), and alkaline phosphatase (ALP) were slightly elevated. The total and direct bilirubin levels were within the normal range in case of all four patients. The triglyceride-glucose (TyG) index is an indicator of insulin resistance [11]. Values greater than 4.49 are indicative of insulin resistance. It is calculated from the values of fasting glucose and fasting triglycerides: TyG = ln (Fasting triglyceride (mg/dl) x Fasting glucose (mg/dl))/2

TyG index values were elevated in all four cases, indicating insulin resistance. Detailed discussion with the patients revealed unhealthy dietary habits and lack of physical activity as the main reasons of overweight/obesity. Table 3 summarizes the daily routine and dietary habits of the patients. All four patients had smart watches that measured the number of steps.

**Table 3:** Physical activity and dietary habits of the subjects before introducing lifestyle changes

Meal/Physical Activity	Time	Details
Breakfast	7.00 am (Summers); 8.00 am (Winters)	2 parathas with omelettes (2 eggs) or kebabs (2) or curry or 2 kebab sandwiches + 1 mug (200 mL) milk
School	7.30–1.20 pm (Summers); 8.30–2.20 pm (Winters)	Routine activity with physical training or games (40 minutes, twice a week)
Mid-morning food break at school	10.45 am (Summers); 11.45 (Winters)	2 parathas with vegetables or 2 stuffed paratha and pickles + 1 fruit
Lunch	1.30 pm (Summers); 2.30 pm (Winters)	3-5 chapatis or 2-3 parathas with chicken/mutton curry or 2 chapatis + 1 bowl rice with vegetables and dal
Games on mobile /Television/Sleep	3.00-4.30 pm	
Evening snacks	4.30-5.00 pm	Tea with 2-3 biscuits or 1 bowl of namkeen or 1 samosa or fritters
Between-meal snacks		Toffees, samosa, fritters, biscuits, cake/muffins
Dinner	8-9.00 pm	3-5 chapatis or 2-4 parathas with chicken/mutton curry or kebab + vegetables or biryani/pulao + 1 sweet dish
Television/mobile	9.00-10.00 pm	
Study	10.00 pm-12.30 am	
Supper	11.30 pm-12.30 am (just before bed)	1 mug milk with flavoring agent and biscuits or 1 kebab sandwich
Total fasting hours	6.5-7 hours at night	
Duration of eating window	16-17 hours	
Total sleep	5-7 hours at night	6-8 hours per 24 hours
Total steps	9000-11000	

The parents were affluent, graduates, but without a science background. Family history revealed a long duration of eating window and late working hours, and a history of hypertension, type 2 diabetes mellitus, renal disease, and cardiovascular events. The fathers of the patients were brothers, aged 45, 42 and 39 years, and were hypertensive and diabetic. Mothers were from different, unrelated families. Both paternal grandparents had died of heart disease in their sixties. One maternal grandfather had died at the age of 58 years due to a heart attack, two maternal grandfathers were hypertensive and diabetic, and one 69-year-old maternal grandmother was diabetic, hypertensive, and on dialysis for end-stage renal disease. The presence of monogenic obesity, hormonal imbalance, etc. was excluded after physical examination and medical history.

Management of the condition

Our aim was to help these patients lose weight, without developing nutritional deficiencies that could affect growth and school performance. Informed consent was taken from the parents before the initiation of the treatment, and for the publication of the results. The patients and their parents also wanted to develop healthy lifestyle habits that would help them keep the weight off. The children needed counselling regarding weight-related teasing that had led them to avoid games and physical activity.

TRE has been tried in adolescent patients with obesity [12]. We obtained parental permission to use this approach and enrolled the patients in a one-year duration study. Since the patients were habituated to their unhealthy lifestyle, we decided to implement changes in a slow and progressive manner. The Specific, Measurable, Attainable, Relevant, and Time-bound; Evidence-based, Strategic, and Tailored to the patient (SMART-EST) goal-setting criteria were followed [13]. No calorie restriction was implemented, however, high calorie foods were omitted or replaced by relatively low-calorie foods. Care was taken to ensure sufficient protein intake from nuts, soybean, gram and pulses, etc. Sleeping hours were extended to seven to eight hours of continuous sleep at night.

Table 4 lists the lifestyle modifications introduced after every 15 days over a period of two months.

**Table 4:** List of lifestyle modifications introduced at 15-day intervals for two months

Chart No.	Duration	Eating window	Diet restrictions implemented	Diet Omissions	Diet substitutions/additions	Physical activity added
1	1-15 days	7 am-11 pm (16 h)	Red meat: once in 15 days	Between-meal snacks	Every meal to be started with a bowl of fruits, sprouts, and vegetables	Half-hour morning walk
2	16-30 days	7 am-10 pm (15 h)	Meat: once in two days	High-calorie fried snacks	Substitute flavored milk with sweetened milk, reduce sugar over weeks	Half-hour of physical activity in the afternoon
3	31-45 days	7 am-8 pm (13 h)	Egg: one per day	Parathas	Roasted gram or wheat/rice puffs as evening snacks	10 minute walk after physical activity in the afternoon
4	46-60 Days	7 am-7 pm (12 h)	Meat: twice a week			Half-hour walk after dinner

The patients were provided the 15-day diet and physical activity charts. The patients were allowed one cheat day during this period. They were asked to report at the end of the 15-day period for further modifications in diet and physical activity.

Outcome and follow-up

Patient A stopped smoking upon being informed about its ill effects, and after his parents came to know about his habit. It must be noted that the young patients had a tough time making the required lifestyle changes, but managed with the support of their parents and other family members. The number of cheat days reduced from two to three per week to two per month at the end of one year. Other members of the extended family stopped eating after 9 pm to reduce the temptations experienced by these adolescents. The patients were advised to further reduce their eating windows to up to 10 hours, continue with the dietary modifications, and increase physical activity.

Anthropometric parameters and laboratory parameters were recorded again after six months and at the end of one year. Table 5 presents the anthropometric parameters and blood pressure of the patients, at the time of initiation of lifestyle modifications, and at the end of six months and one year.

**Table 5:** Anthropometric parameters of the participants at zero, six and 12 months after initiating lifestyle modifications ΔBMI: Decrease in BMI from initial value; WHR: Waist-hip ratio; ΔWHR: Decrease in WHR from initial value; SBP: Systolic blood pressure; DBP: Diastolic blood pressure; 0: Initial reading, at the time of recruitment; 1: Reading after 6 months; 2: Reading after 12 months

Subject	A			B			C			D		
	0	1	2	0	1	2	0	1	2	0	1	2
Height (cm)	172	173	173.5	168	169	171	155.5	156	157	158	160	161.5
Weight (kg)	89	83.5	77.4	75.5	75	72.5	57	56	52.5	62	61	57.5
BMI (kg/m^2^)	30.1	27.9	25.7	26.8	26.3	24.8	23.7	23.0	21.3	24.8	23.8	22.0
ΔBMI		2.2	4.4		0.5	2.0		0.7	1.7		1.0	2.8
Waist (cm)	105	92	90	96.5	91	88.5	87.5	84	83	91	87	85.5
Hips (cm)	107	94	93	100.5	96.5	95	93	92	93	95.5	95	94.5
WHR	0.98	0.98	0.97	0.96	0.94	0.93	0.94	0.91	0.89	0.95	0.92	0.90
ΔWHR		0.00	0.01		0.02	0.03		0.03	0.05		0.03	0.05
SBP (mmHg)	117	117	118	110	110	110	107	106	104	108	108	108
DBP (mmHg)	75	76	75	72	70	72	68	68	68	70	70	70

Fasting glucose, fasting lipid profiles and the TyG index were obtained at the time of initiation of lifestyle modifications, and after six months and one year, and are presented in Table 6.

**Table 6:** Fasting glucose levels and lipid profiles of the participants at zero, six, and 12 months after initiating lifestyle modifications FBG: Fasting blood glucose; T Ch: Total cholesterol; HDL-C: High density lipoprotein-cholesterol; LDL-C: Low density lipoprotein-cholesterol; TG: Triacylglycerols; TyG: Triglyceride-glucose index; 0: Initial reading, at the time of recruitment; 1: Reading after 6 months; 2: Reading after 12 months.

Subject	A			B			C			D		
	0	1	2	0	1	2	0	1	2	0	1	2
FBG (mg/dL)	121.8	112.2	100.8	103.3	97.3	89.7	97.7	94.2	83.1	101.4	97.9	86.4
T Ch (mg/dL)	182.4	175.2	168.8	178.2	165.4	160.3	172.6	164.1	159.2	176.5	167.3	161.2
HDL-C (mg/dL)	37.4	33.5	37.0	38.6	38.2	39.1	48.5	49.5	49.0	47.3	46.9	47.1
LDL-C (mg/dL)	108.2	106.5	98.1	107.8	97.1	92.2	93.6	86.6	83.1	97.5	90.7	85.5
TG (mg/dL)	184.2	176.2	168.5	159.2	150.7	145.2	152.6	140.0	135.4	158.7	148.3	143.2
TyG	5.01	4.95	4.87	4.85	4.80	4.74	4.80	4.74	4.66	4.84	4.79	4.71

HbA1c of patient A decreased from 6.2% to 5.8% after six months, and further decreased to 5.4% at the end of 12 months.

The hemoglobin and values of LFT parameters are presented in Table 7.

**Table 7:** Hemoglobin and liver function test parameters of the patients at zero, six, and 12 months after initiating lifestyle modifications Hb: HemoglobinL; TP: Total protein; Alb: Serum albumin; A/G: Albumin to globulin ratio; AST: Aspartate transaminase; ALT: Alanine transaminase; GGT: Gamma glutamyl transferase; ALP: Alkaline phosphatase; 0: Initial reading, at the time of recruitment; 1: Reading after 6 months; 2: Reading after 12 months.

Subject	A			B			C			D		
	0	1	2	0	1	2	0	1	2	0	1	2
Hb (g/dL)	17.2	15.8	16.8	16.0	16.2	16.0	15.4	15.0	14.6	15.2	16.0	15.4
TP (g/dL)	8.5	8.2	8.2	8.3	8.4	7.8	7.8	7.9	7.4	8.8	8.3	8.3
Alb (g/dL)	4.8	5.0	4.5	5.0	4.8	4.8	4.8	4.9	5.0	5.1	4.9	4.8
A/G	1.3	1.6	1.2	1.5	1.3	1.6	1.6	1.6	2.1	1.4	1.4	1.4
AST (IU/L)	52	49	45	35	33	30	28	22	24	30	27	27
ALT (IU/L)	54	52	45	37	30	30	25	17	16	25	23	19
GGT (IU/L)	41	36	36	38	33	31	23	21	20	25	22	22
ALP (IU/L)	328	314	319	319	302	320	302	312	303	316	319	321

Values of total and direct bilirubin were within the normal range in all cases and have not been presented here.

## Discussion

All four patients had obesity at the time of initiation of the study (Table 1) and were encountering weight-related teasing by peers. Counseling sessions conducted in-person and telephonically involved mitigation of weight-related teasing, elimination of guilt experienced on cheat days, and motivations for physical activity. The adolescents were motivated to play amongst themselves to avoid bullying by their peers. Physical activity was initially increased through walking and subsequently through games. At the end of six months, the patients had started playing badminton and basketball at home and school. None of the participants encountered any problem in increasing physical activity.

Physical examination and the normal levels of hemoglobin, total protein, albumin, and albumin-to-globulin (A/G) ratio in all four cases suggested the absence of nutritional deficiencies. Patient A had FBG and HbA1c in the prediabetic range. All four patients had elevated levels of total serum cholesterol and triglycerides for their age (Table 2). The high-density cholesterol levels were low for age and gender. Although the levels of total and direct bilirubin were normal in all four patients, the levels of liver enzymes were slightly elevated for age and gender, suggesting the risk of future non-alcoholic fatty liver disease (NAFLD) [14].

Introduction of lifestyle modifications was extremely difficult for the adolescents in this study. Their preference of high calorie foods and long duration of eating windows (Table 3) had resulted in excess calorie intake over a period of years. The physical activity level was very low, as they avoided group games at school and at home due to weight-related teasing by their playmates. Our focus was on substitution of high calorie diet with low-calorie options, reducing the eating window to 12 hours, and increase the physical activity. The patients were not asked to check calorie content of foods nor were advised to count calories. Patient A did not face much difficulty in giving up smoking on being persuaded by his family.

These adolescents relished non-vegetarian food, and would often consume different forms at breakfast, lunch, and dinner, and sometimes even during the night. The families agreed to decrease the consumption of meat and eggs and increase the amounts of protein-rich vegetarian foods (pulses, beans, nuts), fresh fruits, and vegetables. Although no calorie restriction had been imposed, the change in food items led to a considerable reduction of calories.

Usually, adults have an average eating window duration of more than 12 hours on week days [15], with the meal pattern varying across countries and across socioeconomic groups [16, 17]. The eating pattern is often erratic on the weekends [18]. The eating window of adolescents has not been studied in detail. Children have a tendency to follow the normal circadian rhythm, however, the transition to adolescence may shift the eating pattern to the adult type. Long eating windows (>14 hours) have been linked to obesity and cardiovascular diseases [18].

The duration of the eating window in case of these four adolescent patients was long: 16-17 hours per day. The eating window started early (7 am in summers and 8 am in winters), due to school timings, and was delayed by 30-60 minutes on Sundays. Implementation of the 12-hours TRE required concluding the last meal of the day at 7 pm. This was extremely difficult, as the adolescents had to study late into the night, and often felt peckish while studying. Initially, the number of diet-related cheat days were two to three per week, however, the number gradually reduced as they became more adept at controlling their food choices and intake. The fact that all four patients were residing in the same house and were eating together helped them motivate each other to adopt the recommended diet changes. 

Lifestyle modifications were introduced in a slow and progressive manner (Table 4), with celebration of each small achievement in the form of praise and (non-food) prize. The patients were encouraged to adopt activities that made them feel happy and forget food, as emotional states and mood are known to influence appetite [19].

The effect of lifestyle modifications on anthropometric parameters recorded at zero, six, and 12 months (Table 5) showed a decrease in BMI and waist-to-hip ratio of all four subjects. The loss of weight or reduction in BMI was not equal in all four cases, this is because of the higher adherence shown by patient A compared to the others. Patients B, C, and D reported a higher number of cheat days. Physical examination and a slight gain in height showed an absence of nutritional deficiencies. Although the subjects were still in the overweight category at the end of 12 months, the parents and the patients reported improvement in the appearance, anthropometric parameters, and boost in confidence. Episodes of bullying and teasing had decreased to almost nil.

FBG and lipid profiles showed improvement in all four cases (Table 6) at the end of one year. Both fasting glucose and HbA1c of patient A were in the normal range at the end of 12 months. The TyG index remained above normal (>4.49) in all four cases at the end of one year. The patients and their parents were informed about the significance of this indicator and were advised to continue with the lifestyle modification program to avoid future fatty liver disease.

Hemoglobin, total protein, albumin, and A/G ratio of the patients remained normal throughout the study period (Table 7). In case of patient A, hemoglobin level decreased from 17.2 to 15.8 at the end of six months. This was probably due to cessation of smoking [20], and not due to nutritional deficiencies. Patient C reported menarche during this period, which may explain the slight decrease in the hemoglobin level.

The levels of non-functional plasma enzymes associated with the liver decreased in all four cases during the study period. High TyG and serum AST, ALT, and GGT have been linked to the metabolic syndrome. Lifestyle modification programs have been linked to the remission of metabolic syndrome [21]. Decreased TyG and decreased levels of AST, ALT, and GGT in the four patients could indicate an improvement in liver health. Since the patients were young, had been diagnosed at a very early stage, and were beginning to develop healthy habits, we are hopeful that they will attain a healthy weight.

The patients have remained in touch and we have observed further improvements in their health status.

Limitations

This case series included only four adolescent patients, who were living in a joint family and were sharing the same meals. Although this was a huge advantage for the patients as it ensured interpersonal motivation and cooperation from all family members, it was a disadvantage for the scientific score of the study in terms of sample size and homogeneity. A larger study of longer duration with a heterogenous sample is required for ensuring the degree of compliance and efficacy of lifestyle modifications in adolescents with overweight or obesity.

## Conclusions

This study highlights the importance of the development of a healthy lifestyle in children. Eating ad libitum, unrestricted calorie consumption, poor choice of food items, and lack of physical activity had resulted in obesity in these adolescents. Slow and progressive changes in their lifestyles were made to ensure the development of healthy and timely eating patterns. Repeat counseling, either face-to-face or telephonically, was required, especially on the days when the patients cheated on their diets.

Since children usually follow the eating patterns and activities of the adults in the family, it is important to develop healthy eating habits at the family level. In this case, the parents and other family members extended their cooperation, and themselves modified their lifestyle to cooperate with the four adolescent members of their family. Calorie restriction was not placed due to the young age of the patients; however, a decrease in the BMI and waist-hip ratio was observed. Repeated counseling sessions with the patients and their parents were required during the study period to ensure cooperation, along with the help of parents and other family members. Early diagnosis of overweight and obesity and timely intervention can help patients achieve a slow and steady weight loss without pharmacotherapy.
